# Low-dose X-ray structure analysis of cytochrome *c* oxidase utilizing high-energy X-rays

**DOI:** 10.1107/S1600577519006805

**Published:** 2019-06-14

**Authors:** Go Ueno, Atsuhiro Shimada, Eiki Yamashita, Kazuya Hasegawa, Takashi Kumasaka, Kyoko Shinzawa-Itoh, Shinya Yoshikawa, Tomitake Tsukihara, Masaki Yamamoto

**Affiliations:** a SR Life Science Instrumentation Team, Life Science Research Infrastructure Group, Advanced Photon Technology Division, RIKEN SPring-8 Center, 1-1-1 Kouto, Sayo-cho, Sayo-gun, Hyogo 679-5148, Japan; b Picobiology Institute, Graduate School of Life Science, University of Hyogo, 3-2-1 Kouto, Kamigori, Akoh, Hyogo 678-1297, Japan; c Institute for Protein Research, Osaka University, 3-2 Yamadaoka, Suita, Osaka 565-0871, Japan; d Protein Crystal Analysis Division, Japan Synchrotron Radiation Research Institute (JASRI), 1-1-1 Kouto, Sayo-cho, Sayo-gun, Hyogo 679-5198, Japan

**Keywords:** radiation damage, high-energy X-ray, macromolecular crystallography

## Abstract

To investigate the effect of high-energy X-rays on site-specific radiation-damage, low-dose diffraction data were collected from radiation-sensitive crystals of the metal enzyme cytochrome *c* oxidase. Data were collected at the Structural Biology I beamline (BL41XU) at SPring-8, using 30 keV X-rays and a highly sensitive pixel array detector equipped with a cadmium telluride sensor. The experimental setup of continuous sample translation using multiple crystals allowed the average diffraction weighted dose per data set to be reduced to 58 kGy, and the resulting data revealed a ligand structure featuring an identical bond length to that in the damage-free structure determined using an X-ray free-electron laser. However, precise analysis of the residual density around the ligand structure refined with the synchrotron data showed the possibility of a small level of specific damage, which might have resulted from the accumulated dose of 58 kGy per data set. Further investigation of the photon-energy dependence of specific damage, as assessed by variations in UV-vis absorption spectra, was conducted using an on-line spectrometer at various energies ranging from 10 to 30 keV. No evidence was found for specific radiation damage being energy dependent.

## Abstract 2

The effect of utilizing high-energy X-rays for macromolecular crystallography of a radiation-sensitive metal enzyme was investigated. The resultant structure was evaluated in combination with visible absorption spectroscopic data. No explicit energy dependence of radiation damage was found in the range from 10 to 30 keV.

## 1. Introduction   

Radiation damage of crystalline specimens caused by incident X-rays in macromolecular crystallography (MX) manifests as degradation of crystal quality (global damage) and local structural changes in molecules (specific damage) as the accumulated dose increases. This is an intrinsic problem associated with the use of highly brilliant light sources at synchrotron facilities, and is undesirable for researchers who wish to complete diffraction data sets or to determine intact molecular structures. Although the absorption of photons by macromolecular crystals, which is proportional to dose, can be decreased by utilization of high-energy (*i.e.* short-wavelength) X-rays, the reduction in diffraction intensity and area-detector efficiency in this energy region presents an obstacle to practical experiments. However, the rate of radiation damage could be reduced by using a sufficiently efficient area detector for high-energy photons. Starting with the Darwin’s formula (Darwin, 1914 ▸, 1922 ▸; Blundell & Johnson, 1976 ▸) for the X-ray diffraction intensity from crystalline samples, Arndt (1984 ▸) showed that the optimal X-ray wavelength for MX could be chosen in terms of the efficiency, which is given by the diffraction intensity divided by the total absorbed photon energy. At least in the X-ray energy range up to 30 keV, efficiency is positively correlated with photon energy, implying that higher energies are better for low-dose data collection. Consistent with Arndt (1984 ▸), Paithankar & Garman (2010 ▸) and Fourme *et al.* (2012 ▸) also suggested that in the energy range between 20 and 40 keV there is an optimal energy for efficient data collection, with detailed estimation taking into account prominent effects for high-energy photons such as Compton scattering and photo-electron escape, as well as protein composition, crystal size and other factors.

In addition to dose-efficiency, Fourme *et al.* (2012 ▸) pointed out that another possible advantage of using high-energy X-rays for MX was the lower experimental errors caused by the lower absorption and the lower extinction effects of crystalline samples. Those effects appear as a reduction in diffraction intensity and the extent is dependent on the X-ray energy, originating from the cross sections of the constituent elements or diffraction quality of the crystalline samples. The linear absorption coefficient of a sample is approximately proportional to the cube of the X-ray wavelength (Helliwell *et al.*, 1993 ▸) and the extinction distance of the incident beam in a crystal is proportional to the X-ray energy (Becker, 1977 ▸). Therefore the use of higher-energy X-rays makes it possible to collect diffraction intensities with smaller errors.

Another issue pertaining to changing the X-ray energies used for MX is the energy dependence of radiation damage, which also has been investigated in a number of pioneering studies. Weiss *et al.* (2005 ▸) investigated site-specific damage by analyzing the decay of difference Fourier peak heights at metal sites of a cadmium derivative of porcine pancreatic elastase using two different wavelengths (1.0 Å and 2.0 Å), and detected no energy dependence. Shimizu *et al.* (2007 ▸) performed a systematic comparison of diffraction intensity statistics of data sets from lysozyme crystals by changing the X-ray energy in nine steps from 6.5 to 33 keV, and concluded that global damage is independent of X-ray energy. Liebschner *et al.* (2015 ▸) also reported that there was no significant difference in global damage to the diffraction intensity of thaumatin crystals in data collected at energies ranging from 6.33 to 19.0 keV. On the other hand, Homer *et al.* (2011 ▸) compared the rate of electron density decay at cysteine sulfurs in lysozyme crystals versus dose, and reported greater specific damage at 14 keV than at 9 keV. Of course, it is difficult to directly compare the results of different studies, some of which reach incompatible conclusions regarding energy dependence, reflecting the fact that different proteins, crystal size and various X-ray energies were used. Particularly in the case of site-specific damage, the phenomenon is dependent on the property of individual sites and environments, including chemical structure, hydro­philicity, and position of the amino acid in the molecule or crystal, making quantitative and uniform evaluation difficult. Therefore, because the results obtained with a particular specimen might not be applicable to another experiment using a distinct protein sample, it would still be worthwhile to characterize the specific radiation damage caused by high-energy X-rays. In any case, it is important to address this issue because data collection efficiency, which is one of the advantages of utilizing high-energy X-rays for MX, could be enhanced or diminished by the X-ray energy-dependence of radiation damage if it occurs.

Bovine heart cytochrome *c* oxidase (CcO) is an enzyme that functions in the cellular respiratory electron transport chain in the mitochondrial inner membrane. CcO accepts four electrons from cytochrome *c* to reduce molecular oxygen (O_2_) to two waters, coupled with the pumping of four protons through the membrane in each catalytic turnover (Yoshikawa & Shimada, 2015 ▸; Wikström *et al.*, 2018 ▸). In its resting oxidized state, it binds a peroxide anion at the O_2_-reduction center, which consists of a copper ion and a heme iron. However, in synchrotron-based MX, the peroxide is readily reduced to water by X-ray irradiation while the diffraction image is recorded, and the bond length between the two oxygen atoms of the peroxide increases as the dose accumulates. To obtain the intact structure, Aoyama *et al.* (2009 ▸) performed a low-dose X-ray diffraction experiment on bovine CcO using a large number of crystals to minimize the X-ray dose absorbed by each one. However, the bond length determined for the bridging ligand, 1.7 Å, was too large for an O—O single bond. Based on the reported experimental conditions, the average diffraction weighted dose (DWD) per data set for this study was estimated as 277 kGy using *RADDOSE-3D* (Zeldin *et al.*, 2013 ▸). The intact crystal structure of the ligand compound was ultimately confirmed as a peroxide by the serial femtosecond rotation crystallography (SF-ROX) method utilizing the XFEL at SACLA (Hirata *et al.*, 2014 ▸).

As suggested by many previous studies, dose-efficient data collection with high-energy X-rays can be practical if a highly efficient area detector for high-energy photons is used. Recently, owing to progress in technologies to apply high-*Z* semiconductors such as cadmium telluride (CdTe) to a single photon counting device, several commercial products applicable to MX have become available. The use of one of these novel detectors for MX, especially for crystal structure analysis of a radiation-sensitive metal enzyme like CcO, will provide the opportunity to address the nature of the energy dependence of specific radiation damage and expand the application of high-energy synchrotron sources.

In this study, we assessed site-specific radiation damage in a radiation-sensitive protein using high-energy X-rays. In particular, low-dose diffraction data were collected from CcO crystals using 30 keV X-rays at BL41XU, SPring-8 (Hasegawa *et al.*, 2013 ▸). A highly sensitive pixel array detector equipped with a CdTe sensor (PILATUS3 X CdTe, DECTRIS) was utilized for data collection by the shutter-less helical scanning method. Maintaining the DWD at 58 kGy per data set by controlling translation-speed and frame-rate settings, we collected a diffraction data set at 1.9 Å resolution (dose estimation with *RADDOSE-3D*). Furthermore, to investigate the possibility of X-ray energy dependence of site-specific damage to the O_2_-reduction center, we applied UV-vis absorption spectroscopy, which sensitively reflects the transient redox state of the heme at this site.

## 2. Methods   

### 2.1. Crystal structure analysis of CcO with 30 keV X-rays   

BL41XU at SPring-8 is a beamline for MX, with high-energy X-rays being available at one of its two end-stations. Utilizing the third harmonic of the undulator, high-energy X-rays ranging from 20 to 35 keV, concentrated by a compound refractive lens (CRL; KIT-IMT), are available at this source. The beam parameters at the sample position used for this study were as follows: horizontal and vertical beam size 0.044 mm × 0.047 mm (top-hat shape assumed for dose calculation); photon flux 8.2 × 10^11^ photons s^−1^; photon energy 30 keV. Usually a CMOS flat-panel detector C10158DK-11 (X) (Hamamatsu Photonics) with a 0.3 mm-thick caesium iodide phosphor is installed, but, at the time this study was conducted, a PILATUS3 X CdTe 300K detector was temporarily available for test use.

**Figure 1:**
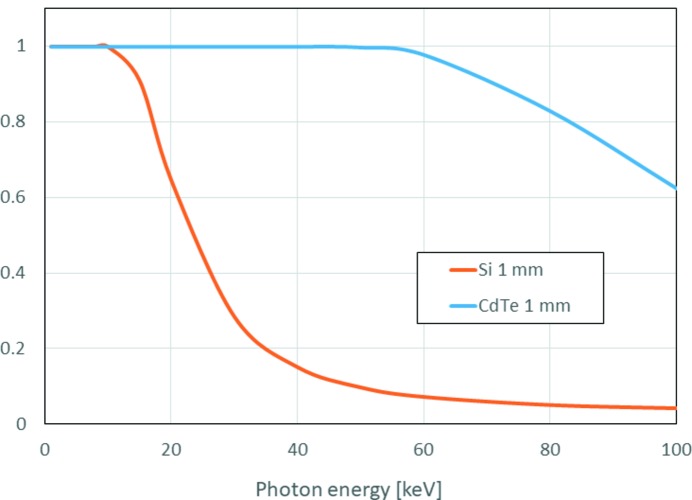
Photon absorption efficiencies of sensor materials. Blue and red lines represent the efficiency of CdTe and Si material with a thickness of 1 mm, respectively. The mass attenuation coefficient was taken from the NIST reference database (NIST, 2004 ▸).

The PILATUS3 X CdTe 300K is a photon-counting pixel array detector with a highly sensitive sensor for high-energy photons consisting of a 1 mm-thick CdTe depletion layer. The pixel dimensions of the horizontal and vertical aperture are 487 × 619 pixels, with a pixel size of 0.172 mm × 0.172 mm. The detector provides a continuous readout of images at a maximum frame rate of 500 Hz (*i.e.* frame acquisition time of 2 ms) with a readout time of 0.95 ms. The photo-absorption efficiency of CdTe is 100% up to 40 keV photon energy, at which conventional silicon sensors of the same thickness yield significantly lower values and are therefore impractical for use (Fig. 1 ▸). Except for the small area size, the specifications of the detector are close to those of an ideal detector for high-energy MX.

In this study, we collected diffraction data under the conditions shown in Table 1 ▸. The shutter-less helical scanning method was applied to large thick crystals of CcO. Because the translation step between each frame was smaller than the beam width, the crystal volume in the footprint of incident beam consisted of a gradation of fresh and damaged portions along the translation axis, at each moment that a diffraction image was recorded. In this case, the dose estimated as the ‘average diffraction weighted dose’ (DWD) by *RADDOSE-3D* is a good description of the dose in the irradiated crystal volume at each moment; this value was 58.0 kGy per data set. Finally, using nine crystals in total, we collected a complete dataset to 1.9 Å resolution (sample to detector distance of 350 mm). A series of helical scanning frame acquisitions was collected by exposing each of the nine utilized crystals from one end to the other with constant rotation around, and translation along, the spindle axis of the horizontal-spindle goniometer. For each crystal, the origin of spindle angle was defined as the broad face of the crystal (typically 0.8 mm × 0.8 mm × 0.2 mm in size, but varying) when it was perpendicular to the incident beam. This makes it possible to align the crystal orientation to the incident beam, because the thin side of a CcO crystal corresponds to the *b*-axis of a unit cell. Typically, a wedge of 30° to 40° diffraction images were collected from a crystal, successively. The starting angle of each helical scanning was defined to continue the previous scanning to cover the hemisphere of the reciprocal space efficiently. If the fresh volume of the crystal was large enough, one or two extra scans were applied after displacing the crystal position perpendicular to the spindle axis so that the irradiated volumes did not overlap with each other. In those cases, the distance of the displacement was determined by considering the width of the footprint of irradiated volume perpendicular to the spindle axis estimated by the function (*V* + *T* sinω)/cosω, where *V*, *T* and ω represent vertical beam size (= 0.047 mm), crystal thickness (= 0.2 mm) and spindle angle, respectively. Because of the small detector size, the detector was vertically offset by 30 mm, and two series of data sets using different horizontal offsets (+35 mm and −35 mm) were collected to cover the required resolution range and multiplicity of diffraction intensities.

The data acquisition conditions, including the exposure time and rotation angle of each frame, were carefully determined to achieve data quality comparable with previous studies (Aoyama *et al.*, 2009 ▸; Hirata *et al.*, 2014 ▸). However, due to limitations on the number of crystals and available beam time, we set the goniometer translation speed (*i.e.* the step between frames) to lower than the maximum value allowed. Consequently, the DWD per data set of 58 kGy was the result of a compromise: if the translation speed had been set ten times higher, and the samples had been exchanged ten times more often, it would have been possible to decrease the DWD per data set to 5.8 kGy in order to complete the dataset.

In total, 4020 diffraction images were processed and merged with *XDS* and *XSCALE* (Kabsch, 2010 ▸). Data collection conditions, along with data processing and refinement statistics, are shown in Table 2 ▸. The initial phases of structure factors up to 4.0 Å resolution were determined by the rigid-body refinement method with *REFMAC* (Murshudov *et al.*, 2011 ▸), using the structure of ligand-free fully reduced CcO previously determined at 1.6 Å resolution [Protein Data Bank (PDB) ID: 5b1b] as the initial model. We chose the structure of a ligand-free protein in a different redox state as a search model to eliminate the model bias in the result of the calculation. The phases were extended to 1.9 Å resolution by the density modification method (Wang, 1985 ▸) coupled with non-crystallographic symmetry averaging using the CCP4 (Collaborative Computational Project, Number 4, 1994 ▸) program *DM* (Winn *et al.*, 2011 ▸). The electron density map with minimized model bias was calculated using the resultant phases. Structure refinement was conducted with *REFMAC*. Bulk solvent correction and the anisotropic scaling of the observed and calculated structure amplitudes were incorporated into the refinement. Anisotropic temperature factors for the iron, copper and zinc atoms were imposed on the refinement model. The quality of the structure refinement was evaluated based on the *R* and *R*_free_ values. Other than the O_2_-reduction center, no substantial changes were detected relative to the previously reported structure (Aoyama *et al.*, 2009 ▸; Hirata *et al.*, 2014 ▸). The coordinates and structure factors were deposited in the PDB with PDB-ID 6j8m.

### 2.2. Visible absorption spectroscopy   

As reported previously (Aoyama *et al.*, 2009 ▸), X-ray irradiation of the CcO crystal (photon energy of 13.8 keV) induces changes in the UV-vis absorption spectrum. The absorption increases at 604 and 582 nm are assignable to reduction of heme a and formation of a low-spin ferrous heme a_3_, respectively, even at cryogenic temperatures. Also the absorption band in the vicinity of 650 nm which can be seen only in the resting oxidized state of CcO is assignable to the existence of high-spin ferric heme a_3_, and decreases as dose accumulates. Therefore, the change in the UV-vis absorption spectrum as a function of absorbed dose can be used as an indicator of the radiation-induced heme reduction/ligand exchange, which reflects specific radiation damage at the O_2_-reduction center during an MX experiment. A number of structural biologists have shown that metal centers of proteins are sensitive to X-ray radiation, and that their absorption spectra change rapidly before a complete diffraction dataset can be collected (*e.g.* Matsui *et al.*, 2002 ▸; Beitlich *et al.*, 2007 ▸; Pearson *et al.*, 2007 ▸; Hough *et al.*, 2008 ▸; McGeehan *et al.*, 2009 ▸; Owen *et al.*, 2009 ▸). Accordingly, on-line micro-spectrometers have often been implemented as a complementary method to X-ray crystallography in order to investigate specific radiation damage, especially for radiation-sensitive specimen such as metal enzymes. This method is applicable to detecting changes that do not appear in the electron density map of crystal structures, and should therefore be useful for assessing the energy dependence of site-specific damage at the O_2_-reduction center of CcO.

To test this idea, we conducted on-line spectroscopy measurements at 30 keV X-ray energy by setting up a portable spectrometer at BL41XU. Incident and detective fiber optics for visible light were set perpendicular to the X-ray beam at the optic center of the diffractometer. The focus diameter of the visible light was 0.05 mm (1/e width of Gaussian shape), smaller than the X-ray beam size of 0.2 mm × 0.2 mm (square) with a photon flux of 7.4 × 10^11^ photons s^−1^. The parallel beam, without a CRL, was attenuated by insertion of a 1 mm-thick aluminium plate, and a top-hat beam shape was assumed for dose calculation using *RADDOSE-3D*. The CcO crystal size was 0.6 mm × 0.6 mm × 0.2 mm. Under these conditions, the dose rate for CcO was estimated as 1.13 kGy s^−1^. This setup allowed us to alternate between measurements of absorption spectra with X-ray irradiation by rotating the crystal goniometer spindle back and forth by 90°. The details of this apparatus were reported previously (Aoyama *et al.*, 2009 ▸). In addition, to evaluate the energy dependence of the spectral changes, we conducted spectroscopic measurements at another beamline, BL44XU (Higashiura *et al.*, 2016 ▸), at the X-ray energies of 10, 13.8 and 17.7 keV. The beam at BL44XU was focused by horizontal and vertical mirror optics, shaped with a 0.05 mm-diameter pinhole device upstream of the sample, and attenuated with aluminium plates. The dose rate for each energy, calculated assuming top-hat shape for the beam, was 1.50, 1.36 and 0.95 kGy s^−1^, respectively. These three measurements at BL44XU were conducted using one crystal with dimensions 0.5 mm × 0.5 mm × 0.15 mm at three different irradiation points to minimize the sample dependence as much as possible.

### 2.3. Preparation of CcO crystals   

CcO in the resting oxidized state was purified from bovine heart mitochondria and crystallized as described previously (Tsukihara *et al.*, 1995 ▸; Mochizuki *et al.*, 1999 ▸). To stabilize crystals, the crystals were soaked in ‘stabilization solution’ containing 40 m*M* sodium phosphate buffer, pH 6.5, 0.2% (*w*/*v*) *n*-decyl-β-d-maltoside, 1%(*w*/*v*) PEG 4000, and 2%(*v*/*v*) ethyl­ene glycol. Then the crystals were flash cooled in a cryo-nitro­gen stream at 100 K after soaking in a solution containing 40 m*M* sodium phosphate, pH 5.7, 0.2% decyl maltoside, 5% PEG 4000, and 45% (*v*/*v*) ethyl­ene glycol as a cryo-protectant. The final medium composition was obtained by 50 (or 40) stepwise *in situ* manual exchange of the stabilization solution with 50 (or 40) different soaking solutions containing steadily increasing ethyl­ene glycol concentrations.

## 3. Results and discussion   

### 3.1. Low-dose (58 kGy) structure of ligand peroxide   

**Figure 2:**
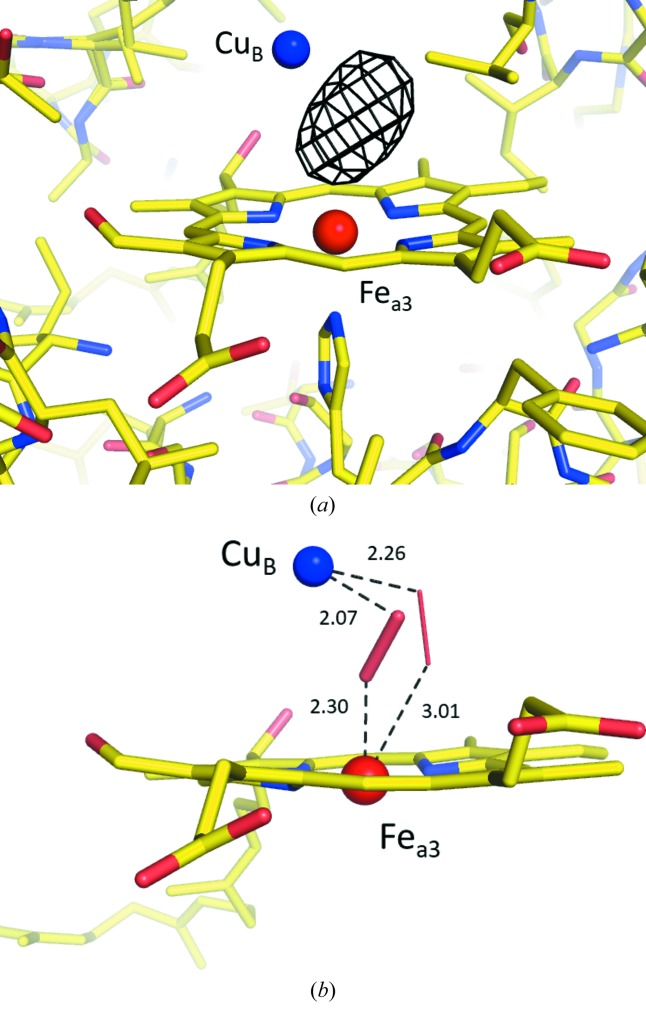
*F*_o_ − *F*_c_ map and ball-and-stick structure model around the binuclear O_2_-reduction center consisting of Cu_B_ (blue) and heme Fe_a3_ (red), with and without a ligand peroxide model. (*a*) *F*_o_ − *F*_c_ difference peaks (black mesh), depicted at the 10σ level (1σ = 0.064 e Å^−3^). (*b*) Structural model with ligand peroxide of 1.55 Å bond length, consisting of 90% major (thick red stick) and 10% minor (thin red stick) components. The difference map is depicted at the 2.5σ level (1σ = 0.063 e Å^−3^). Numerical values adjacent to the peroxide show the distances in angstroms (Å).

**Figure 3:**
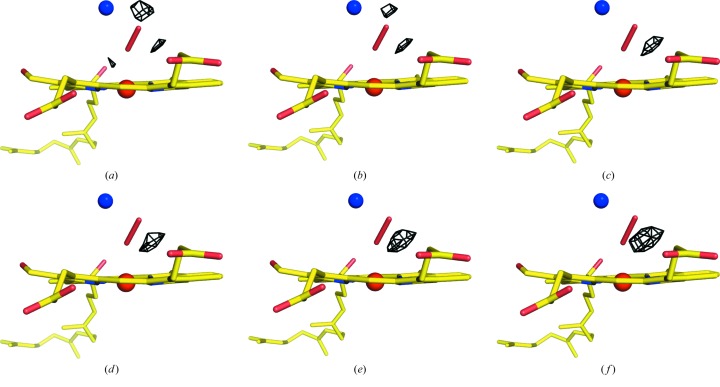
Evaluation of the most accurate bond length of the peroxide ligand (red stick) at the O_2_-reduction center. Structure refinement was carried out with a fixed bond length, which was varied from 1.45 to 1.70 Å in steps of 0.05 Å. Panels (*a*), (*b*), (*c*), (*d*), (*e*) and (*f*) correspond to the fixed bond lengths of 1.45, 1.50, 1.55, 1.60, 1.65 and 1.70 Å, respectively. *F*_o_ − *F*_c_ difference peaks (black mesh) are depicted at the 2.5σ level (1σ = 0.063 e Å^−3^).

Fig. 2 ▸ shows the structure around the O_2_-reduction center of CcO refined to 1.9 Å resolution based on the structure-factor amplitudes obtained with 30 keV X-rays. Without a ligand model, the *F*_o_ − *F*_c_ difference map shows an elliptical residual peak between the copper (Cu_B_) and heme a_3_ iron (Fe_a3_) [Fig. 2(*a*) ▸]. The ligand model was refined, focusing on its bond length, following the procedure adopted in the damage-free structure analysis of SF-ROX (Hirata *et al.*, 2014 ▸) and of Aoyama *et al.* (2009 ▸). Structure refinement with the ligand model, without any restraint on bond length between the two oxygen atoms, yielded a bond length of 1.66 Å, shorter than in previous work in which refinement was performed in a similar manner. When different refinements were started with the initial ligand model having shorter and longer distances of 1.55 and 1.85 Å independently, both cases converged to the same bond length of 1.66 Å. The *F*_o_ − *F*_c_ map contained a large residual peak next to the peroxide. The ligand bond length was further evaluated by fixing it during the refinement, and then varying it from 1.45 to 1.7 Å in increments of 0.05 Å to find the optimal value for describing the structure that yielded the optimal residual density map (Fig. 3 ▸). When the bond length of the model was too short, residual density peaks appeared on the outer side of each oxygen atom. Conversely, longer bond lengths enhanced the residual peak next to the bond. Ultimately, the optimal bond length was determined to be 1.55 Å, which did not give large residual peaks neither outside nor beside the bond. This was identical to that in the damage-free structure obtained with SF-ROX (Hirata *et al.*, 2014 ▸). Finally, the residual density was minimized by defining multiple conformer models with a 10% minor component of peroxide, similar to the structure analysis by SF-ROX, which used a 5% minor component (Hirata *et al.*, 2014 ▸) [Fig. 2(*b*) ▸].

Table 3 ▸ summarizes the results of the structure refinement in comparison with published work (Aoyama *et al.*, 2009 ▸; Hirata *et al.*, 2014 ▸). When inspecting fixed and refined bond lengths of peroxide, our result regarding the specific damage is comparable with that of the damage-free SF-ROX (Hirata *et al.*, 2014 ▸) structure. Thus 30 keV X-rays and a CdTe pixel array detector are feasible for data collection at a dose low enough to identify the intact chemical structure of the radiation-sensitive ligand of CcO.

### 3.2. Energy dependence of the visible absorption spectrum   

**Figure 4:**
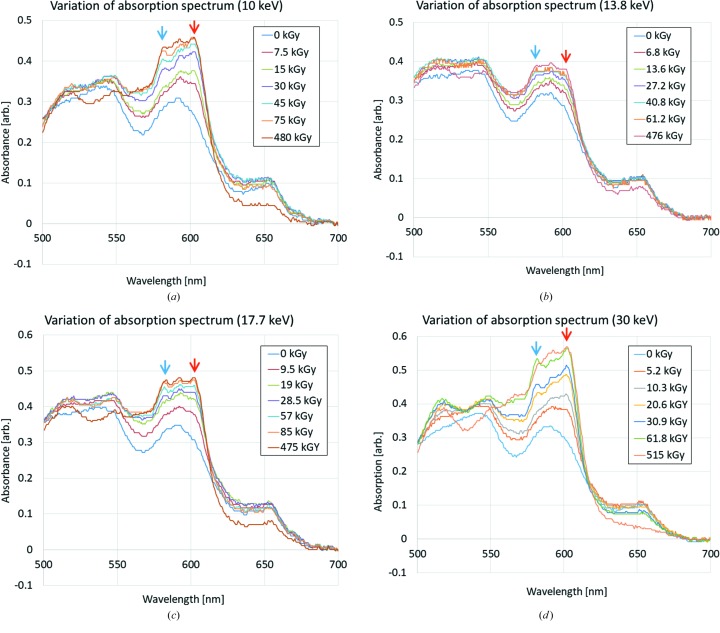
Variation in UV-vis absorption spectra of CcO crystals in the resting oxidized state as a function of absorbed dose, measured following irradiation with (*a*) 10, (*b*) 13.8, (*c*) 17.7 and (*d*) 30 keV X-rays, respectively. Red and blue arrows indicate characteristic peak growths versus dose at wavelengths of 604 and 582 nm, respectively.

**Figure 5:**
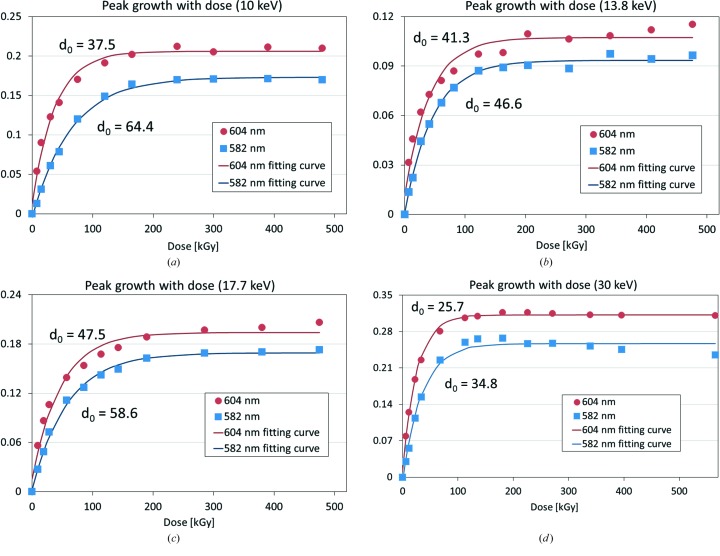
Peak height growths in visible absorption spectra at 604 nm (solid circles) and 582 nm (solid squares), relative to the isosbestic point of 630 nm, versus dose following irradiation with (*a*) 10, (*b*) 13.8, (*c*) 17.7 and (*d*) 30 keV X-rays, respectively. Exponential functions (red and blue solid lines), with the exponential constants [*d*_0_ (kGy)] shown in the figures, were fitted to the data.

The visible absorption spectra as a function of dose, measured at four different X-ray energies (10, 13.8, 17.7 and 30 keV), are shown in Fig. 4 ▸. At all energies, variations in absorption profile with increasing dose were accompanied by characteristic peak growths at two wavelengths, 604 and 582 nm, as indicated in the figure, and a decrease of the 650 nm peak at high dose. As described above, these changes represent evidence of reduction of heme a and formation of a low-spin ferrous heme a_3_ and they were confirmed to occur similarly at all X-ray energies. To investigate further, the increases in peak heights relative to the absorbance at the isosbestic point of 630 nm versus dose are plotted in Fig. 5 ▸. At all energies, the peaks began to grow at a very early stage of dose accumulation and reached their maximum level at 80–150 kGy. The growth rates of these peak heights are considered to be proportional to the fraction of heme a and heme a_3_ active sites in the crystal that can change the redox state in response to X-ray irradiation at constant dose rate. Thus, the dose dependence of the peak growth should follow the law of exponential growth, which is analogous to the analysis reported in previous work (Matsui *et al.*, 2002 ▸; Borshchevskiy *et al.*, 2014 ▸). In those studies, the authors analyzed the dose dependence of the absorption spectrum change originated from X-ray-induced generation of orange species in bacteriorhodopsin crystals caused by specific damage to the bound retinal. In our case, the function of [*a*_0_ − *a*_1_ exp(−*d*/*d*_0_)] was fitted to the peak-growth plots by the least-squares method, where *d* represents dose, and *a*_0_, *a*_1_ and *d*_0_ are fitting parameters (Fig. 5 ▸).

**Figure 6:**
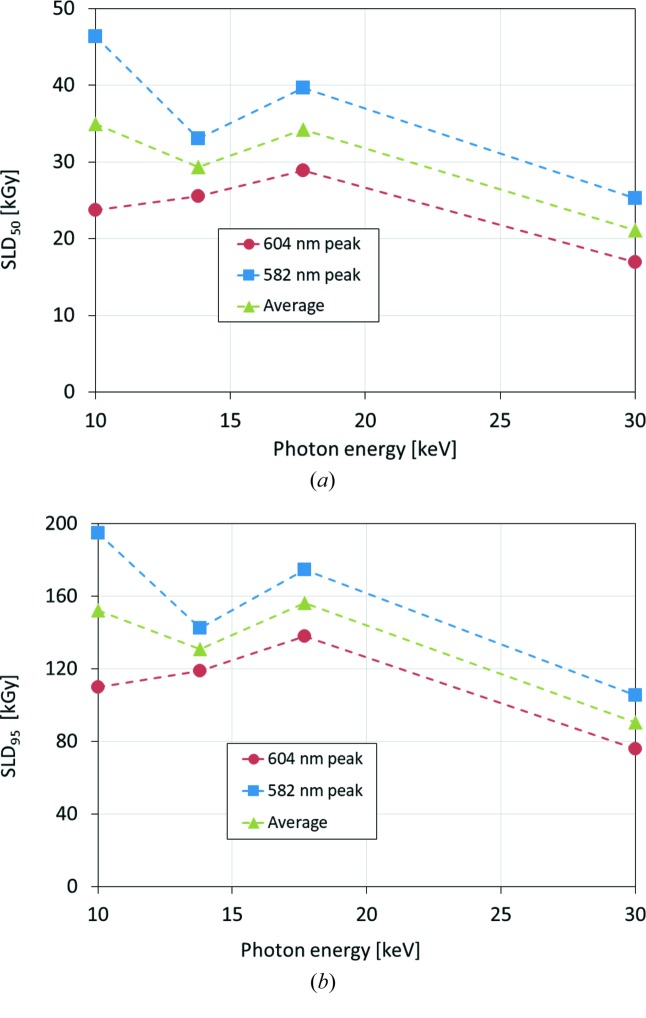
Variation in spectroscopic lifedoses (*a*) SLD_50_ and (*b*) SLD_95_ versus X-ray energies of 10, 13.8, 17.7 and 30 keV. For each panel, SLDs were estimated from 582 nm peak (solid squares), 604 nm peak (solid circles) and the average of those values (solid triangles) are plotted.

The fitted curve was further analyzed to estimate the relationship between growth rate and dose, by introducing the dose limitation termed ‘spectroscopic lifedose’ (Hersleth & Andersson, 2011 ▸), hereafter SLD, which corresponds to the dose at which peak growth reaches 50% (SLD_50_ or half lifedose) or 95% (SLD_95_) of the maximum value. The SLDs estimated from the plots in Fig. 5 ▸ are summarized in Table 4 ▸, and the variation versus photon energy is plotted in Fig. 6 ▸. In all cases, SLD_50_ and SLD_95_ were of the order of tens to hundreds of kGy, significantly smaller than the experimental dose limit for MX at cryo-temperature; for example, 30000 kGy for the averaged diffraction intensity to decay to 0.7 of its original value (Owen *et al.*, 2006 ▸). For the 30 keV data measured at BL41XU, the SLDs had the smallest values out of all the X-ray energies, whereas the SLDs for the other three energies (10, 13.8 and 17.7 keV) all measured at BL44XU using the same crystal, yielded larger SLD values. For these three energies, the variance in the SLDs was small, and the largest SLD_50_ and SLD_95_ values were obtained at 10 and 17.7 keV, respectively, whereas the smallest SLDs were obtained at the intermediate energy of 13.7 keV. Hence, at least from these results, there seems to be no consistency in the energy dependence of the SLDs.

The difference in the SLDs for 30 keV relative to the other three energies may be the result of differences in the experimental conditions, considering the fact that the measurements at other energies were conducted using a different crystal sample at another beamline with different beam properties. Thus, the results are not suitable for direct comparison. In particular, at BL44XU, the X-ray beam was cut with a 0.05 mm pinhole upstream of the sample, which was comparable with the size of the visible-light beam used for spectroscopy. Therefore, it is possible that the visible absorption profiles measured at BL44XU were affected by bleeding of visible light around the circumference of the X-ray beam, and that the SLDs were overestimated. However, it is obvious that the use of high-energy X-rays at 30 keV did not yield any spectroscopic evidence that specific damage was suppressed.

## 4. Conclusions   

In this study, we performed dose-efficient diffraction data collection at 30 keV incident X-ray energy for the radiation-sensitive metal protein CcO in the resting oxidized state, which is suggested by theoretical arguments to be appropriate for the use of high-energy X-rays for MX. This was achieved by introducing a highly efficient area detector equipped with CdTe sensor material. This experimental setup enabled us to collect a complete dataset at a low dose (58 kGy) up to a resolution of 1.9 Å, comparable with the data quality (resolution, completeness, multiplicity *etc*.) in former studies on CcO. The ligand peroxide structure was obtained without elongation by specific radiation damage; the bond length of 1.55 Å was comparable with that of the damage-free structure obtained using an XFEL. The final refinement model of CcO converged to a 10% minor component of ligand peroxide at the O_2_-reduction center, larger than the 5% minor component of the XFEL structure. This difference is likely to represent the structural change caused by specific radiation damage on the structure obtained in the current study using a finite exposure time at the synchrotron source. If this is the case, the existence of a 5% minor component in the XFEL structure raises the question of whether specific damage was also suffered by that structure. In the SF-ROX method, it is possible that radical diffusion in-between successive XFEL shots caused specific damage, even when the crystal has been translated between XFEL pulses. To clarify this, a further investigation with structure determination in the absence of any influence from neighboring data collections is required, for instance, SF-ROX with a single XFEL shot for each crystal. In any case, the diffraction data and the resulting crystal structure we obtained enabled identification of the intact chemical structure of the radiation-sensitive ligand of CcO, indicating that this approach to diffraction data collection with 30 keV X-rays was both practical and useful for MX.

*RADDOSE-3D* estimates the dose-efficiency for a certain diffraction condition and this is output as the ‘Diffraction Efficiency’ parameter, which corresponds to the elastic yield of diffracted photons divided by the average diffraction weighted dose, and the value for the conditions used in this study for CcO (58 kGy DWD per data set at 30 keV X-ray) was 1.1 × 10^9^ photons kGy^−1^. In comparison, the value calculated under otherwise identical conditions at an X-ray energy of 13.8 keV is 6.8 × 10^8^ photons kGy^−1^. In addition, the value calculated for a lower energy of 10 keV is 5.2 × 10^8^ photons kGy^−1^. This means that the gain in dose-efficiency using 30 keV X-rays versus 13.8 and 10 keV is about 1.6- and 2.1-fold, respectively. Although this increase is not dramatic, the use of higher-energy X-rays significantly benefits researchers intending to collect a complete dataset from multiple crystals, while minimizing the X-ray dose and reducing the number of crystals required. In general, a well known advantage of utilizing high-energy X-rays at MX beamlines is that it enables ultra-high resolution structure analysis. However, with the application of high-*Z* detectors for such experiments, it will also become possible to use high-energy X-rays for low-dose diffraction data collection, and our ability to obtain intact structures of radiation-sensitive proteins will increase.

By using complementary UV-vis absorption spectroscopy, we observed sensitive variations in the spectra versus dose at all energies from 10 to 30 keV. We obtained no explicit evidence of the merit of using 30 keV X-rays to suppress specific radiation damage. Therefore, we concluded that the result of MX at 30 keV which yielded the short bond length for the ligand peroxide, was not the consequence of the X-ray energy itself, but rather of the low-dose data collection strategy. Furthermore, the comparison of spectroscopic results using identical crystals at BL44XU yielded no evidence that the spectrum change was energy-dependent, at least not at energies ranging from 10 to 17.7 keV.

In this study, the estimated diffraction weighted dose per data set for MX was 58 kGy, still larger than the spectroscopic lifedose (SLD_50_) at 30 keV. It would be worth conducting an MX study at a lower dose than this value in order to investigate the relationship between spectroscopy and crystal structures at low doses. As mentioned, it would be possible to further decrease the dose by increasing the translation speed of the crystal goniometer during data collection. Specifically, the dose would decrease in inverse proportion to the translation speed, without a reduction of data quality. However, the number of crystals required to complete the data would increase proportionally to the translation speed. In this study, if it had been possible to use a larger area detector (*e.g.* PILATUS3 X CdTe 1M), it would not have been necessary to offset the detector position. Then the data collection efficiency, regarding the number of scan series to cover the hemisphere of the reciprocal space, and the number of crystals, would have been better. The application of high-energy X-rays is expected to be further enhanced by the use of novel detectors with improved specifications.

## Supplementary Material

PDB reference: bovine heart cytochrome c oxidase, 6j8m

## Figures and Tables

**Table 1:** Conditions for diffraction data collection from CcO crystals

Beamline	BL41XU (endstation 1), SPring-8
Photon energy	30 keV
Photon flux	8.2 × 10^11^ photons s^−1^
Beam size	0.044 mm × 0.047 mm (horizontal and vertical)
Temperature	100 K
Area detector	PILATUS3 X CdTe 300K
Detector distance	350 mm
Detector offset	Vertical: +30 mm; horizontal: ±35 mm
Crystal size	0.8 mm × 0.8 mm × 0.2 mm
Exposure time	0.25 s per frame (continuous readout in 4 Hz)
Rotation step	0.1° per frame (continuous rotation)
Translation step	2.3 µm per frame (continuous horizontal translation)
Average DWD per data set†	58.0 kGy
Total number of images	4020
Total number of crystals	9

† Average diffraction weighted dose calculated with *RADDOSE-3D*.

**Table 2:** Statistics for reflections and structure refinement of CcO at a dose of 58 kGy (DWD per data set) Structure refinement was performed with the multi-component ligand peroxide model having a fixed bond length of 1.55 Å and a 10% minor component.

Data collection		
Space group		*P*2_1_2_1_2_1_
Cell dimensions of *a*, *b*, *c* (Å)		182.02 (0.28)†, 203.54 (0.22)†, 177.41 (0.28)†
Resolution (Å)		40.00–1.90 (1.97–1.90)‡
Observed reflections		2466011 (207563)‡
Averaged multiplicity		4.90 (4.23)‡
〈*I*/σ(*I*)〉		8.1 (1.4)‡
Completeness (%)		98.1 (96.3)‡
*R*_merge_		0.099 (0.973)‡
*R*_pim_		0.047 (0.535)‡
CC_1/2_		0.994 (0.697)‡
		
Refinement		
Resolution (Å)		40.00–1.90
No. of reflections (all/free)		503298 / 25030
*R* / *R*_free_		0.172 / 0.199
No. of atoms		
	Protein	29021
	Ligand / ion	2314
	Water	2558
Average *B* (Å^2^)		
	Protein	39.9
	Ligand / ion	70.3
	Water	51.6
RMS deviations (Å)		
	Bond lengths (Å)	0.014
	Bond angles (°)	1.59
Ramachandran plot statistics (%)		
	Favored regions	96.4
	Allowed regions	3.1
	Disallowed regions	0.52

† Estimated standard deviation.

‡ Value of the highest resolution shell.

**Table 3:** Comparison of the ligand structure refinement of CcO in the resting oxidized state, based on datasets collected at different X-ray energies

Intensity data		BL41XU SPring-8†	BL3 SACLA‡	BL44XU SPring-8§
Photon energy (keV)		30	10	13.8
Resolution (Å)		40.0–1.9	27.3–1.9	40.0–1.95
*R* / *R*_free_		0.172 / 0.199	0.195 / 0.230	0.181 / 0.208
Ligand peroxide bond length (Å)				
	Refined	1.66	1.75	1.90
	Fixed	1.55	1.55	1.70

† Intensity datasets obtained in this work.

‡ Intensity datasets obtained by Hirata *et al.* (2014 ▸).

§ Intensity datasets obtained by Aoyama *et al.* (2009 ▸).

**Table 4:** Spectroscopic life-dose (SLD) of CcO O_2_-reduction center in the resting oxidized state, estimated from the UV-vis absorption spectra. Spectroscopic life-doses (SLD_50_ and SLD_95_) correspond to 50% and 95% of maximum peak growth of 582 and 604 nm, respectively

		Crystal 1						Crystal 2	
		Beamline BL44XU						Beamline BL41XU	
Energy (keV)		10 keV		13.8 keV		17.7 keV		30 keV	
SLD (kGy)		SLD_50_	SLD_95_	SLD_50_	SLD_95_	SLD_50_	SLD_95_	SLD_50_	SLD_95_
	604 nm peak	23.7	110.0	25.5	119.0	28.9	138.2	16.9	76.0
	582 nm peak	46.4	194.8	33.1	142.6	39.7	174.6	25.3	105.4
	Average	35.0	152.4	29.3	130.8	34.2	156.4	21.1	90.7

## References

[bb1] Aoyama, H., Muramoto, K., Shinzawa-Ito, K., Hirata, K., Yamashita, E., Tsukihara, T., Ogura, T. & Yoshikawa, S. (2009). *Proc. Natl Acad. Sci.* **106**, 2165–2169. 10.1073/pnas.0806391106 PMC2650126 19164527

[bb2] Arndt, U. W. (1984). *J. Appl. Cryst.* **17**, 118–119.

[bb3] Becker, P. (1977). *Acta Cryst.* A**33**, 243–249.

[bb4] Beitlich, T., Kühnel, K., Schulze-Briese, C., Shoeman, R. L. & Schlichting, I. (2007). *J. Synchrotron Rad.* **14**, 11–23. 10.1107/S0909049506049806 17211068

[bb5] Blundell, T. L. & Johnson, L. N. (1976). *Protein Crystallography.* New York: Academic Press.

[bb6] Borshchevskiy, V., Round, E., Erofeev, I., Weik, M., Ishchenko, A., Gushchin, I., Mishin, A., Willbold, D., Büldt, G. & Gordeliy, V. (2014). *Acta Cryst.* D**70**, 2675–2685. 10.1107/S1399004714017295 25286851

[bb7] Collaborative Computational Project, Number 4 (1994). *Acta Cryst.* D**50**, 760–763.

[bb8] Darwin, C. G. (1914). *Philos. Mag. Ser. 6*, **27**, 315–333.

[bb9] Darwin, C. G. (1922). *Philos. Mag. Ser. 6*, **43**, 800–829.

[bb10] Fourme, R., Honkimäki, V., Girard, E., Medjoubi, K., Dhaussy, A.-C. & Kahn, R. (2012). *J. Appl. Cryst.* **45**, 652–661.

[bb11] Hasegawa, K., Shimizu, N., Okumura, H., Mizuno, N., Baba, S., Hirata, K., Takeuchi, T., Yamazaki, H., Senba, Y., Ohashi, H., Yamamoto, M. & Kumasaka, T. (2013). *J. Synchrotron Rad.* **20**, 910–913. 10.1107/S0909049513022176 PMC3795554 24121338

[bb12] Helliwell, J. R., Ealick, S., Doing, P., Irving, T. & Szebenyi, M. (1993). *Acta Cryst.* D**49**, 120–128. 10.1107/S0907444992006747 15299553

[bb13] Hersleth, H.-P. & Andersson, K. K. (2011). *Biochim. Biophys. Acta*, **1814**, 785–796. 10.1016/j.bbapap.2010.07.019 20691815

[bb14] Higashiura, A., Yamashita, E., Yoshimura, M., Hasegawa, K., Furukawa, Y., Kumasaka, T., Ueno, G., Yamamoto, M., Tsukihara, T. & Nakagawa, A. (2016). *AIP Conf. Proc.* **1741**, 030028.

[bb15] Hirata, K., Shinzawa-Itoh, K., Yano, N., Takemura, S., Kato, K., Hatanaka, M., Muramoto, K., Kawahara, T., Tsukihara, T., Yamashita, E., Tono, K., Ueno, G., Hikima, T., Murakami, H., Inubushi, Y., Yabashi, M., Ishikawa, T., Yamamoto, M., Ogura, T., Sugimoto, H., Shen, J., Yoshikawa, S. & Ago, H. (2014). *Nat. Methods*, **11**, 734–736. 10.1038/nmeth.2962 24813624

[bb16] Homer, C., Cooper, L. & Gonzalez, A. (2011). *J. Synchrotron Rad.* **18**, 338–345. 10.1107/S0909049511005504 PMC3083911 21525641

[bb17] Hough, M. A., Antonyuk, S. V., Strange, R. W., Eady, R. R. & Hasnain, S. (2008). *J. Mol. Biol.* **378**, 353–361. 10.1016/j.jmb.2008.01.097 18353369

[bb18] Kabsch, W. (2010). *Acta Cryst.* D**66**, 125–132. 10.1107/S0907444909047337 PMC2815665 20124692

[bb19] Liebschner, D., Rosenbaum, G., Dauter, M. & Dauter, Z. (2015). *Acta Cryst.* D**71**, 772–778. 10.1107/S1399004715001030 PMC4388262 25849388

[bb21] McGeehan, J., Ravelli, R. B. G., Murray, J. W., Owen, R. L., Cipriani, F., McSweeney, S., Weik, M. & Garman, E. F. (2009). *J. Synchrotron Rad.* **16**, 163–172. 10.1107/S0909049509001629 PMC2651762 19240328

[bb20] Matsui, Y., Sakai, K., Murakami, M., Shiro, Y., Adachi, S., Okumura, H. & Kouyama, T. (2002). *J. Mol. Biol.* **324**, 469–481. 10.1016/s0022-2836(02)01110-5 12445782

[bb22] Mochizuki, M., Aoyama, H., Shinzawa-Itoh, K., Usui, T., Tsukihara, T. & Yoshikawa, S. (1999). *J. Biol. Chem.* **274**, 33403–33411. 10.1074/jbc.274.47.33403 10559221

[bb23] Murshudov, G. N., Skubák, P., Lebedev, A. A., Pannu, N. S., Steiner, R. A., Nicholls, R. A., Winn, M. D., Long, F. & Vagin, A. A. (2011). *Acta Cryst.* D**67**, 355–367. 10.1107/S0907444911001314 PMC3069751 21460454

[bb37] NIST (2004). NIST X-ray Mass Attenuation Coefficients, NIST Standard Reference Database 126. National Institute of Standards and Technology, Gaithersburg, MD, USA (doi:10.18434/T4D01F).

[bb24] Owen, R. L., Pearson, A. R., Meents, A., Boehler, P., Thominet, V. & Schulze-Briese, C. (2009). *J. Synchrotron Rad.* **16**, 173–182. 10.1107/S0909049508040120 PMC2651763 19240329

[bb25] Owen, R. L., Rudiño-Piñera, E. & Garman, E. F. (2006). *Proc. Natl Acad. Sci.* **103**, 4912–4917. 10.1073/pnas.0600973103 PMC1458769 16549763

[bb26] Paithankar, K. S. & Garman, E. F. (2010). *Acta Cryst.* D**66**, 381–388. 10.1107/S0907444910006724 PMC2852302 20382991

[bb27] Pearson, A. R., Pahl, R., Kovaleva, E. G., Davidson, V. L. & Wilmot, C. M. (2007). *J. Synchrotron Rad.* **14**, 92–98. 10.1107/S0909049506051259 17211075

[bb28] Shimizu, N., Hirata, K., Hasegawa, K., Ueno, G. & Yamamoto, M. (2007). *J. Synchrotron Rad.* **14**, 4–10. 10.1107/S0909049506049296 17211067

[bb29] Tsukihara, T., Aoyama, H., Yamashita, E., Tomizaki, T., Yamaguchi, H., Shinzawa-Itoh, K., Nakashima, R., Yaono, R. & Yoshikawa, S. (1995). *Science*, **269**, 1069–1074. 10.1126/science.7652554 7652554

[bb30] Wang, B. C. (1985). *Methods Enzymol.* **115**, 90–112. 10.1016/0076-6879(85)15009-3 4079800

[bb32] Weiss, M. S., Panjikar, S., Mueller-Dieckmann, C. & Tucker, P. A. (2005). *J. Synchrotron Rad.* **12**, 304–309. 10.1107/S0909049505003328 15840915

[bb33] Wikström, M., Krab, K. & Sharma, V. (2018). *Chem. Rev.* **118**, 2469–2490. 10.1021/acs.chemrev.7b00664 PMC6203177 29350917

[bb34] Winn, M. D., Ballard, C. C., Cowtan, K. D., Dodson, E. J., Emsley, P., Evans, P. R., Keegan, R. M., Krissinel, E. B., Leslie, A. G. W., McCoy, A., McNicholas, S. J., Murshudov, G. N., Pannu, N. S., Potterton, E. A., Powell, H. R., Read, R. J., Vagin, A. & Wilson, K. S. (2011). *Acta Cryst.* D**67**, 235–242. 10.1107/S0907444910045749 PMC3069738 21460441

[bb35] Yoshikawa, S. & Shimada, A. (2015). *Chem. Rev.* **115**, 1936–1989. 10.1021/cr500266a 25603498

[bb36] Zeldin, O. B., Gerstel, M. & Garman, E. F. (2013). *J. Appl. Cryst.* **46**, 1225–1230.

